# Inflammatory cytokines and neurotrauma biomarkers are acutely elevated in military combat casualties and predict resource use

**DOI:** 10.3389/femer.2026.1906777

**Published:** 2026-08-16

**Authors:** Nathan H. Johnson, Nancy G. Casanova, Sara M. Camp, Leopoldo Cancio, Yansong Li, Joe G. N. Garcia

**Affiliations:** 1 Center for Inflammation Science and Systems Medicine, University of Florida Scripps Research Institute, Jupiter, FL, United States,; 2 US Army Burn Center, US Army Institute of Surgical Research, JBSA Fort Sam Houston, TX, United States,; 3 Department of Expeditionary Critical Care Research, US Army Institute of Surgical Research, JBSA Fort Sam Houston, TX, United States

**Keywords:** DAMP, eNAMPT, polytrauma, TC3, TLR4

## Abstract

**Introduction:**

Triage of and evacuation decisions for military service members deployed into combat zones experiencing traumatic injuries are serious challenges for military medical providers. We explored the utility of inflammatory biomarkers in determining injury severity and predicting hospital resource utilization to improve the effectiveness of casualty care.

**Methods:**

Serum levels of inflammatory cytokines (eNAMPT, IL-6, PSGL-1, ANG-2, IL-1β, IL-1RA, TNF-α, and S100A8) and neurotrauma biomarkers (GFAP, NfL, and tau) were analyzed in trauma-exposed warfighters (*n* = 39) evacuated to a medical treatment facility. Prior to analysis, outliers were removed via robust regression and outlier removal, with the results analyzed using the Mann–Whitney test, Kruskal–Wallis test w/ Dunn, or Spearman correlation.

**Results:**

All the tested biomarkers were significantly elevated (*p* < 0.0001) in combat casualties vs. healthy controls (*n* = 46) and exhibited injury type-specific elevations. Serum eNAMPT (*p* = 0.033) and TNF-α (*p* = 0.047) were significantly higher in penetrating wounds vs. blunt trauma; IL-1β (*p* = 0.005) and S100A8 (*p* = 0.005) were significantly elevated in thermal injures vs. blunt trauma; and GFAP (*p* < 0.001) and NfL (*p* = 0.017) were significantly elevated in blunt trauma vs. thermal injury. Statistical correlations between biomarkers and clinical outcomes indicated the following: (i) duration of mechanical ventilatory support correlated with IL-1RA level (*p* = 0.021); (ii) duration of hospital stay correlated with IL-6 (*p* = 0.016) and eNAMPT levels (*p* = 0.046); and (iii) duration of ICU stay correlated with eNAMPT (*p* = 0.016) and S100A8 (*p* = 0.017) levels.

**Conclusions:**

Our results extend prior reports of innate immune activation following traumatic injury and suggest a diverse panel of inflammatory biomarkers may predict hospital resource utilization to inform prioritization of local resources and evacuation to higher levels of care.

## Introduction

Triage of and evacuation decisions for military service members deployed into combat zones who experience traumatic injuries (blunt force, explosion, or gunshot), and a subsequent heightened risk of critical illness, are serious challenges for military medical providers. In the civilian sphere, ambulances and emergency medical technicians are able to, on average, stabilize patients in 14 min on scene and transport them to an appropriate higher level of care in 11 min (1). In the military sphere, however, the response time can vary significantly depending on the tactical environment and the resources available. In combat situations, the need to gain fire superiority or secure an evacuation site can result in a delay in emergency medical care for combat casualties (2). In theatre, line units can have combat medics assigned as part of their operational team. Combat medics are trained in advanced prehospital life support techniques and can directly respond to combat-induced injury or environmentally imposed illness in the field. Utilizing Tactical Combat Casualty Care (TC3), combat medics can secure, stabilize, and prepare a casualty for transport to a higher echelon of care (2, 3). In instances of reduced supply availability or mass-casualty events, TC3 provides guidelines on how to effectively screen and triage casualties to make the most effective use of limited medical supplies and best determine the evacuation priority of each casualty (2, 4). The accurate determination of a casualty’s evacuation priority is paramount, as an underclassification could lead to an additional delay of care or evacuation to a lower-level treatment facility, while an overclassification can result in the unnecessary waste of finite medical resources in the field or clinical environment (5). As such, there is a need for additional tools that allow military medics or physicians to better determine the severity of a casualty’s injuries, forecast the amount of resources needed for treatment, and predict the probable prognostic outcome.

The physiological response to trauma involves the release of damage-associated molecular pattern (DAMP) proteins by destroyed or damaged cells, which induce inflammation through activation of the innate immune system (6, 7). Consistent with other published studies (8–13), we recently reported the prognostic value of serum biomarkers of innate immunity dysregulation in traumatic brain injury (TBI)-exposed subjects and their utility in assessing the severity of bidirectional lung–brain axis injuries (9). In the current study, we postulated that this panel of innate immunity biomarkers may discriminate the type of injury in combat warfighters and facilitate triage decision-making. Serum measurements of systemic inflammatory cytokines/DAMPS (eNAMPT, IL-6, PSGL-1, ANG-2, IL-1β, IL-1RA, TNF-α, and S100A8) and neurocentric biomarkers [glial fibrillary acidic protein (GFAP), neurofilament light chain (NfL), and total tau] from combat casualties revealed a diverse panel of inflammatory biomarkers that may predict hospital resource utilization and inform prioritization of local resources and evacuation to settings providing a higher level of care.

## Methods

### Patient sample collection and classification

The patient samples consisted of blood serum isolated from whole blood drawn from US Army combat casualties in the Iraqi theatre or from a historical cohort of healthy controls. Blood was drawn immediately after medical evacuation to a military medical treatment facility or field hospital. Combat casualties were classified as having either blunt, penetrating, or thermal trauma by military medical personnel. Samples were isolated and stored at −80°C until analysis.

### Serum biomarker analysis

Patient serum was analyzed for concentrations of inflammatory DAMPs (eNAMPT and S100A8), cytokines (IL-1β, IL-6, IL-1RA, and TNF-α), vascular markers (Ang-2 and PSGL-1), and cellular structure proteins (GFAP, NfL, and total tau) using the Meso Scale Discovery (MSD; MD, USA) R-Plex and U-Plex platform as we have previously described (9). Briefly, serum was thawed and diluted at a 1:4 ratio prior to addition to the plate. Plates were incubated at room temperature for 1 h on a plate shaker prior to being placed at 4°C overnight. The following day, the plates were washed and incubated in secondary detection antibodies for 1 h on a plate shaker at room temperature while protected from light. The plates were then washed and read solution was added. Plates were immediately read on the MSD Quick-Plex instrument. Raw data were interpreted utilizing MSD Discovery Workbench software and Microsoft Excel. Two MSD plates were used with the R-Plex plate for measurements of GFAP, NfL, S100A8, and total tau, and the remainder utilized the U-Plex plate.

### Statistical methods

All samples were analyzed utilizing GraphPad Prism software. Samples were assigned to groups according to injured vs. control and type of injury. Prior to analysis, all samples and groups were analyzed for statistical outliers using robust regression and outlier removal (ROUT) analysis with a Q set to 1% (14). After the removal of statistically identified outliers, sample groups were compared using the non-parametric two-tailed Mann–Whitney test or Kruskal–Wallis test with *post-hoc* Dunn correction. A receiver operating characteristic (ROC) curve was calculated to determine each individual biomarker’s overall sensitivity and specificity to trauma vs. healthy controls. A Spearman correlation analysis was performed to test the correlation between the biomarkers and clinical measures. When combining data from all injury types to assess clinical indicators, patients with missing data relating to the clinical indicator of interest were omitted.

## Results

### Patient demographics

Patient samples were drawn from US Army combat casualties (*n* = 39, average 41.6 ± 3 years old, 27 male, 4 female, and 8 unreported) from the Iraqi theatre or from a historical cohort of healthy controls (*n* = 46, average 53 ± 2 years old, 11 male, 8 female, and 27 unreported). Injury groups consisted of 14 blunt, 5 penetrating, and 12 thermal trauma casualties, of which 26 required an average of 12.8 ± 3.1 days of mechanical ventilation and spent an average of 39.6 ± 6 days in the hospital and 20.4 ± 3.7 days in the ICU. Eight patients had missing phenotypic data related to their injury or whether they received any specific hospital care, and the reported overall mortality was 8%, with no cases of sepsis reported (Table 1).

### Significant elevations in systemic and neurocentric innate immunity biomarkers in combat casualties

Previous studies in clinical and preclinical models of trauma have demonstrated the utility of inflammatory cytokines, DAMPs, and cell structure proteins in determining the severity of the traumatic insult and probable prognostic outcome (8, 9, 15, 16). In this study, all tested biomarkers were significantly elevated (*p* < 0.0001) in combat casualties compared to healthy controls (Figure 1). ROC analysis of the tested biomarkers exhibited the following area under the curves for the individual biomarkers: total tau, 1.0; IL-1RA, 1.0; IL-1β, 1.0; IL-6, 1.0; S100A8, 0.99; Ang-2, 0.98; eNAMPT, 0.97; NfL, 0.95; GFAP, 0.94; PSGL-1, 0.89; and TNF-α, 0.88; all with a *p*-value of <0.0001 (Figure 2).

### Variation in serum biomarker levels in combat casualties by type of combat injury

Previous clinical studies have demonstrated that, in addition to the severity of an injury, the types of biomarkers that are elevated after trauma can also vary depending on the type of insult (9, 17). When comparing biomarkers to injury types, eNAMPT [mean (M): 37.65, SEM: 13.66, *p* = 0.033] and TNF-α (M: 3.18, SEM: 0.36, *p* = 0.047) were significantly higher in penetrating wounds compared to blunt trauma (M: 10.53, SEM: 1.08 and M: 2.06, SEM: 0.13). IL-1β (M: 1.77, SEM: 0.30, *p* = 0.005) and S100A8 (M: 13,836, SEM: 2,502, *p* = 0.005) were significantly elevated in thermal injures compared to blunt trauma (M: 0.74, SEM: 0.12 and M: 4,962, SEM: 593.2). GFAP (M: 20,297, SEM: 5,026, *p* < 0.001) and NfL (M: 261.6, SEM: 36.66, *p* = 0.017) were significantly elevated in blunt trauma compared to thermal (M: 297.4, SEM: 66.15 and M: 122.6, SEM: 21.84), with total tau (M: 99.54, SEM: 19.33, *p* = 0.020) elevated in blunt trauma compared to penetrating injury (M: 36.60, SEM: 8.79) (Figure 3).

### Point of injury biomarker levels correlate with clinical resource use

Our previous studies have demonstrated that inflammatory cytokines and DAMP levels in patient serum were correlated with ICU stay and mechanical ventilation utilization (9). We next assessed the utility of our innate immunity biomarker panel in predicting clinical resource utilization based on baseline measurements after trauma. We observed correlations between baseline IL-1RA level (*r*: 0.47, *p* = 0.021) and the need for and duration of mechanical ventilatory support. The total duration of hospital admission positively correlated with IL-6 (*r*: 0.49, *p* = 0.016) and eNAMPT levels (*r*: 0.39, *p* = 0.046). Finally, the duration of ICU stay, which would require the most resources and specialized equipment, positively correlated with eNAMPT (*r*: 0.47, *p* = 0.016) and S100A8 (*r*: 0.46, *p* = 0.017) levels (Figure 4).

## Discussion

In this study, we demonstrate that the tested systemic inflammatory cytokines/DAMPs and neurocentric biomarkers are significantly elevated in the serum of military casualties acutely after combat-related injury. Furthermore, we demonstrate how the tested biomarkers vary depending on injury classification, identifying key biomarkers that could be utilized to better classify polytrauma casualties. After trauma, DAMPs are released from dead or damaged cells to bind pathogen recognition receptors and activate innate immunity, inducing the production of inflammatory cytokines (18). eNAMPT and S100A8 are DAMPs that function as innate immunity sentinels via binding and activation of the TLR-4/NF-kb pathway (19, 20). In our combat cohort, casualties exhibited significantly elevated levels of both tested DAMPs after injury. Interestingly, when comparing between injury types, eNAMPT was highest in penetrating injury while S100A8 was highest in thermal injury when both were compared to blunt force trauma. eNAMPT has been well-documented in lung injury and has also been shown clinically to be a major participant in sepsis pathobiology (13, 21). Our results reinforce previous studies reporting S100A8 elevation in a preclinical inhalation burn model and in patients with dermal burns (22, 23). We further observed that casualties exhibited significantly elevated levels of each tested inflammatory cytokine (IL-6, IL-1β, IL-1RA, and TNF-α) when compared to healthy controls, findings in alignment with previous studies in clinical and preclinical models that demonstrated that inflammatory cytokines are significantly elevated post-trauma (8, 9, 17, 21, 24).

Neuropathological biomarkers, such as NfL, GFAP, and total tau, can be effectively used to determine neurotrauma severity and the progression of neurodegeneration (25). However, evidence is emerging that these traditionally central nervous system-associated biomarkers are also active and can originate from pathologies in peripheral organs. GFAP is a monomeric filament cytoskeletal protein that is strongly associated with activated astrocytes, and clinical studies have correlated elevated GFAP levels with worsened CT results and negative prognostic outcomes after TBI (25–27). NfL is a component of myelinated axons, which are primarily expressed in white matter, and has been shown to be an effective biomarker of axonal injury in TBI (25). Tau is a microtubule-associated protein that is important in the formation and maintenance of the cytoskeleton of axons. It has been shown to be an effective biomarker of repeated TBIs in athletes and is also associated with neurodegeneration in Alzheimer’s disease (6, 25). In our combat cohort, all three neurotrauma biomarkers were elevated in casualties compared to healthy controls. Additionally, when comparing between injury types, all three biomarker levels were higher in blunt force trauma compared to either penetrating or thermal trauma. Considering that injuries such as TBI and spinal cord injury would most likely be classified as blunt force trauma, it is not surprising that these neurotrauma biomarkers are more elevated in this casualty type.

Finally, in this study, we showed the utility of systemic inflammatory cytokines/DAMPs and neurocentric biomarkers in patient triage, and that acute measurement of serum biomarkers can predict a casualty’s eventual clinical resource utilization, including the duration of ICU stay and use of mechanical ventilation. Early indicators of poor patient outcomes can be used by clinicians to make the most informed decisions when it comes to care. Previous studies have demonstrated the utility of inflammatory cytokines, DAMPs, and neurotrauma biomarkers in determining the probable outcomes for a critically ill or injured patient (8, 13, 28, 29). We performed three specific measurements to determine the correlation between acute biomarker levels and total number of days spent in the hospital, the duration of ICU stay, and number of days requiring mechanical ventilation. When assessing which biomarkers correlated with the number of days spent in the hospital or ICU, IL-6 and eNAMPT levels were both moderately and positively correlated with a casualty’s total hospital stay, while eNAMPT and S100A8 correlated with duration of ICU stay. Considering that eNAMPT and S100A8 can both trigger IL-6 expression via TLR4, and that elevated eNAMPT is reported in numerous trauma pathologies, we speculate a more severe injury would induce greater inflammatory responses and require more care (9, 13, 30). IL-1RA and TNF-α were both positively correlated with the need for and duration of mechanical ventilation, consistent with studies linking these proteins to trauma and lung injury (9, 13, 21, 31).

Despite these insights into the potential utility of polytrauma biomarkers in predicting additional resource use, several limitations of the current study need to be recognized. These include the relatively small cohort size and the complication of missing phenotypic data related to demographics, injury, or clinical outcomes. Although the type of injury each casualty sustained was reported, data on the specific regions impacted, the specific cause of injury, or if the casualty had any preexisting comorbidities or previous injuries were not available. An additional limitation is that, whereas these biomarkers can be readily assayed in a well-equipped medical laboratory setting, not all these biomarkers are available in point-of-care testing. However, recent research has demonstrated efficacy in testing biomarkers such as GFAP and NfL in blood spot testing, which could allow for the testing of these biomarkers in a field-expedient manner (32). Finally, considering that these casualties came from one theatre of combat, future studies will need to investigate the potential for environmental impacts that could alter the results.

In conclusion, our biomarker panel composed of systemic inflammatory cytokines/DAMPs and neurocentric biomarkers is effective in predicting which patients will need the most clinical resources and provider attention. Additionally, we demonstrate that biomarker levels are injury-specific and could potentially be utilized to better diagnose which organ systems are impacted in polytrauma cases. Future studies will need to investigate these biomarkers in larger casualty cohorts and in cohorts with additional clinical and demographic data on specific injury diagnosis and personal demographics. Furthermore, with recent research demonstrating the utility of blood spot testing in measuring neurotrauma biomarkers, there is potential for the development of field-expedient assays that could be utilized with a minimal amount of blood or need for venipuncture. Utilizing these biomarkers, forward operating role one medical providers could have additional data to better triage combat casualties by more accurately determining a casualty’s evacuation priority and the appropriate level of care earlier, allowing for optimized local resource utilization.

## Figures and Tables

**FIGURE 1:**
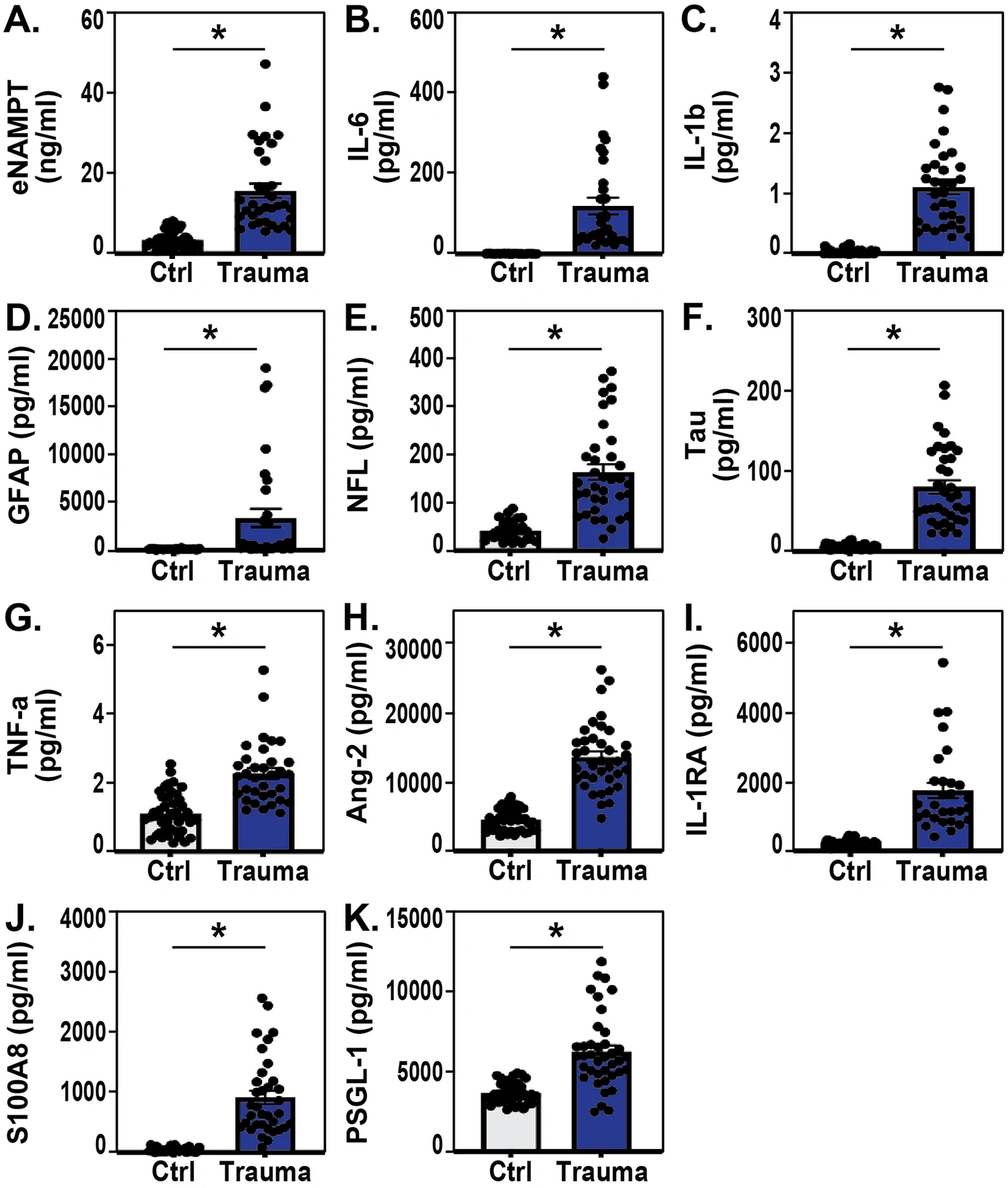
There are significant elevations in systemic and neurocentric innate immunity biomarkers in trauma-exposed combat casualties. Biomarker levels were measured in serum of combat casualties at the time of baseline injury. All biomarkers are significantly elevated (*p* < 0.0001) in combat casualties **(A–K)**. Outliers were removed via ROUT analysis, then analyzed using two-tailed Mann–Whitney tests. Data are shown as mean ± SEM.

**FIGURE 2:**
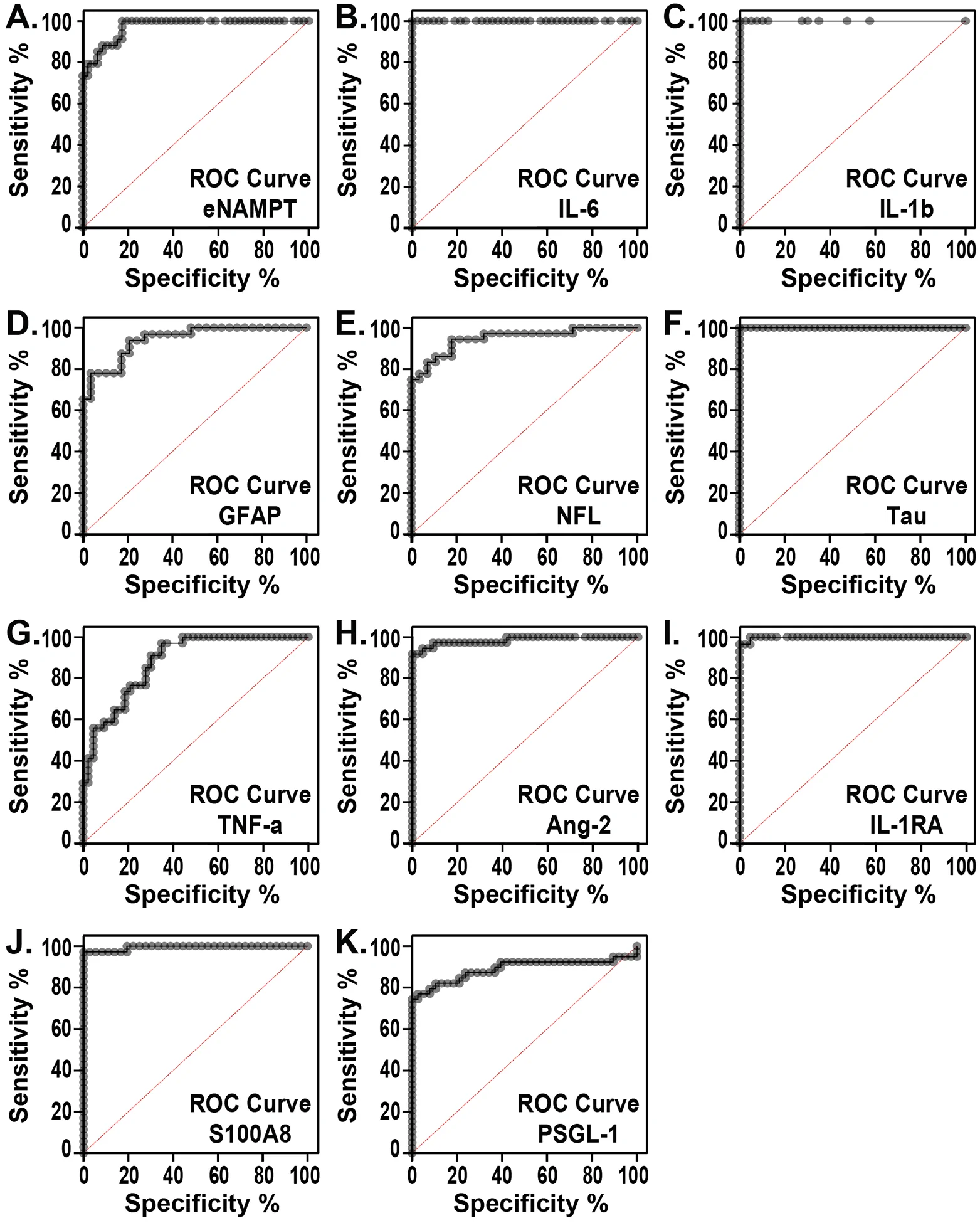
Neuro and innate immunity proteins are effective serum biomarkers of polytrauma in combat casualties. The ROC analysis demonstrates significant sensitivity for detecting trauma in each tested biomarker in combat casualties compared to healthy controls **(A–K)**. Area under the curve for each biomarker is as follows: total tau, 1.0; IL-1RA, 1.0; IL-1β, 1.0; IL-6, 1.0; S100A8, 0.99; Ang-2, 0.98; eNAMPT, 0.97; NfL, 0.95; GFAP, 0.94; PSGL-1, 0.89; and TNF-α, 0.88; all with a *p*-value of <0.0001. Outliers were removed via ROUT analysis prior to ROC analysis.

**FIGURE 3:**
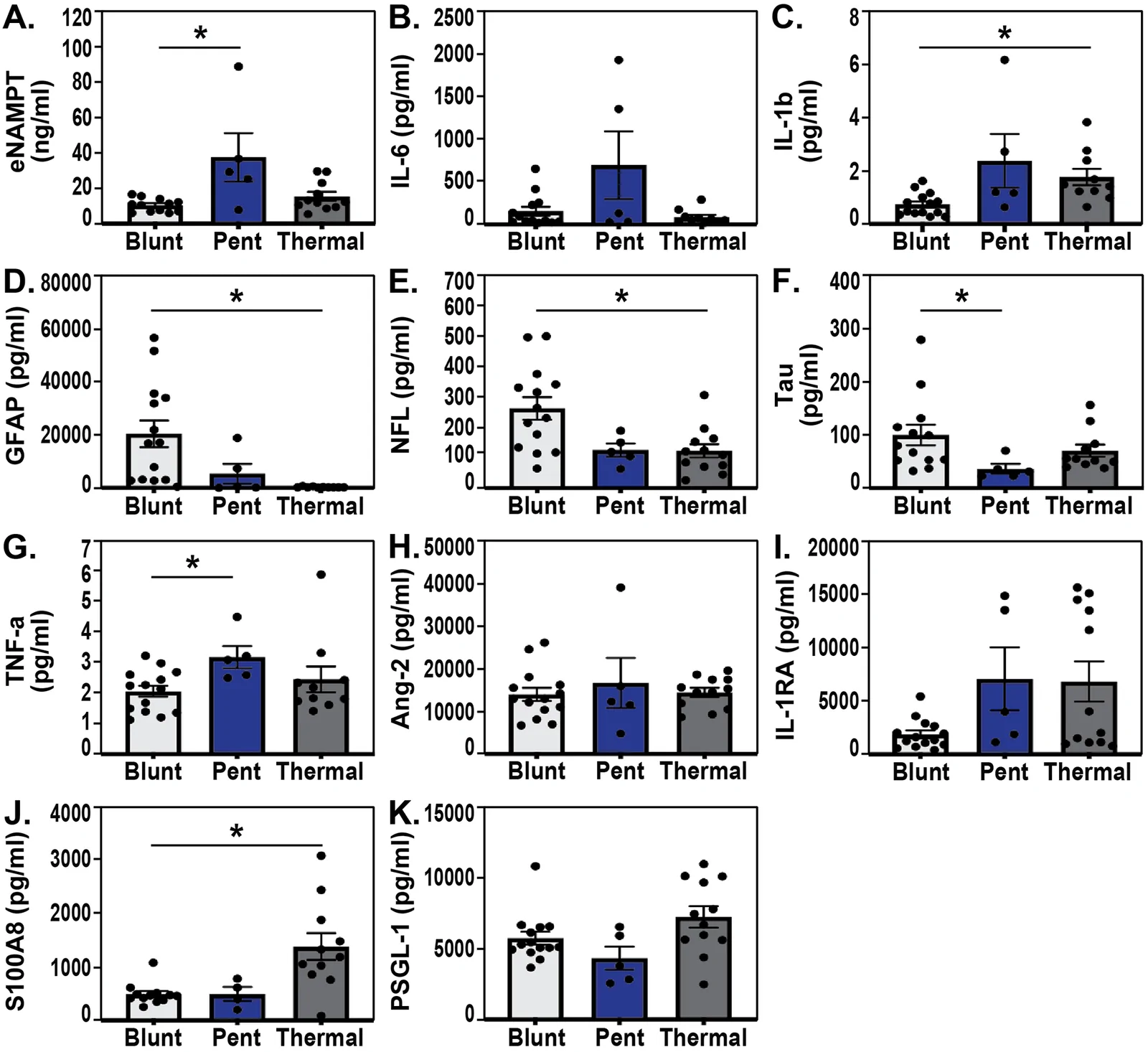
Neurocentric and innate immunity biomarker levels are linked to injury classification. **(A–K)** Serum biomarker levels were assessed for their association with trauma injury type, such as penetrating wounds compared to blunt trauma (eNAMPT, TNF-α), thermal injury compared to blunt trauma (IL-1β, S100A8), blunt trauma compared to thermal injury (GFAP, NfL), and blunt trauma compared to penetrating injury (total tau). Outliers were removed via ROUT analysis and then analyzed using the Kruskal–Wallis test w/ *post-hoc* Dunn correction. Data are shown as mean ± SEM.

**FIGURE 4:**
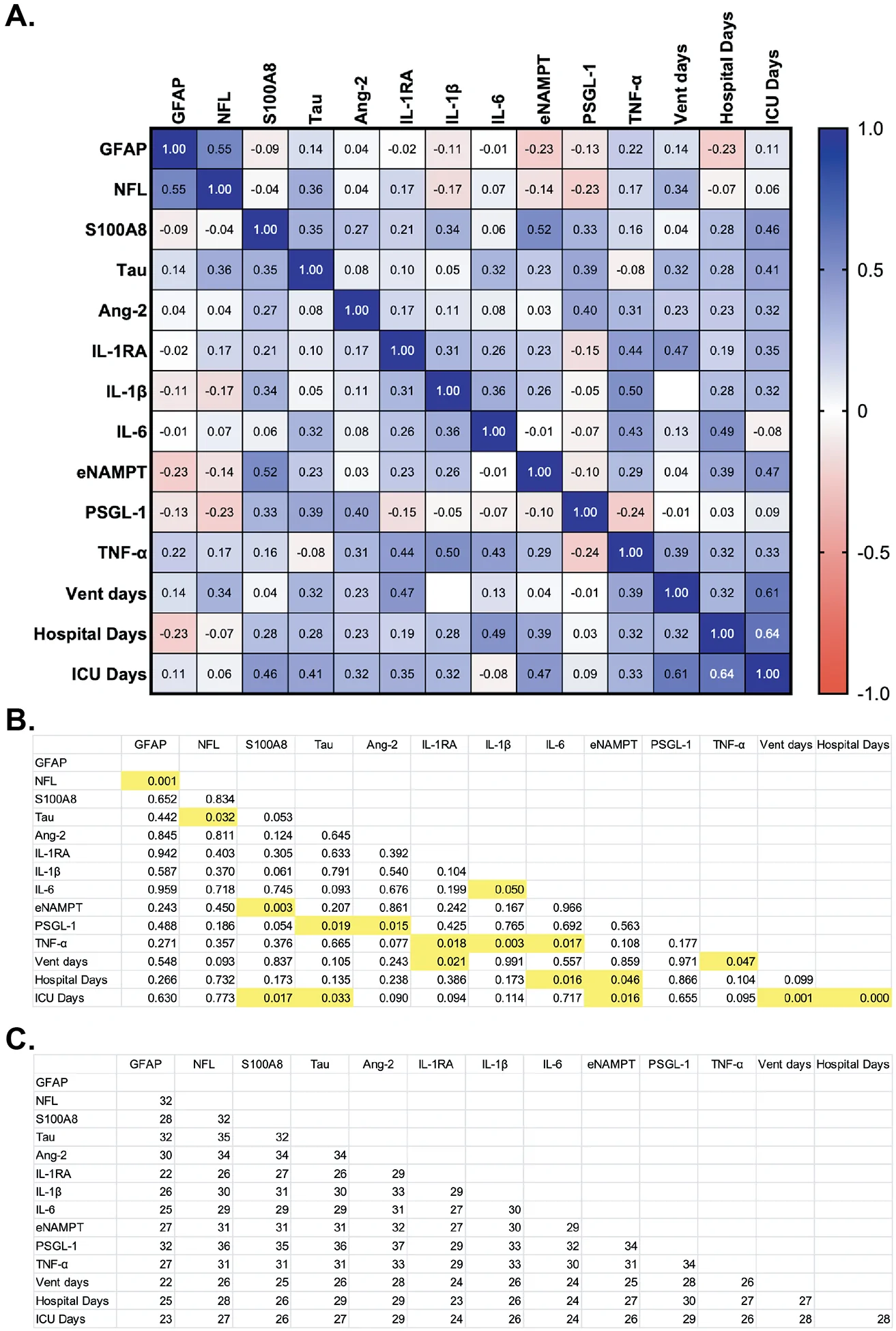
Correlation of neurocentric and innate immunity biomarker levels with duration of hospital stay and resource use. Spearman correlation of all the tested biomarkers with *q*-values shown **(A)**. Serum eNAMPT level is significantly correlated with duration of hospital (*p* = 0.046) and ICU (*p* = 0.016) stays, while IL-1RA (*p* = 0.021) and TNF-α (*p* = 0.047) levels are correlated with duration of mechanical ventilation. Significant (*p* < 0.05) values are highlighted in yellow **(B)**. *N*-values for each group after ROUT analysis **(C)**.

**Table 1:** Patient demographics by cohort. M: male, F: female, U: unknown. Data presented as Mean+/− SEM.

Cohort Demographics			
Type	n Value	Average Age (years)	Sex
Healthy	46	53 +/− 2	M11/F8/U27
Trauma	39	41.6 +/− 3	M27/F4/U8
Injury Groups		Resource Utilization	
Type	n Value	Type	Average Days
Blunt	14	Mechanical Ventilation	12.8 +/− 3.1
Penetration	5	Hospital	39.6 +/− 6
Thermal	12	ICU	20.4 +/− 3.7

## Data Availability

The raw data supporting the conclusions of this article will be made available by the authors, without undue reservation.
